# Prevalence and correlation of cytokine-specific autoantibodies with epidemiological factors and C-reactive protein in 8,972 healthy individuals: Results from the Danish Blood Donor Study

**DOI:** 10.1371/journal.pone.0179981

**Published:** 2017-06-30

**Authors:** Jakob Hjorth von Stemann, Andreas Stribolt Rigas, Lise Wegner Thørner, Daniel Guldager Kring Rasmussen, Ole Birger Pedersen, Klaus Rostgaard, Christian Erikstrup, Henrik Ullum, Morten Bagge Hansen

**Affiliations:** 1 Department of Clinical Immunology, Rigshospitalet, Copenhagen University Hospital, Copenhagen, Denmark; 2 Department of Clinical Immunology, Næstved Sygehus, Næstved, Denmark; 3 Epidemiology research, Statens Serum Institut, Copenhagen, Denmark; 4 Department of Clinical Immunology, Aarhus University Hospital, Aarhus, Denmark; Katholieke Universiteit Leuven Rega Institute for Medical Research, BELGIUM

## Abstract

Natural cytokine-specific autoantibodies (c-aAb) have been measured in healthy and diseased individuals, and have been considered as both endogenous immune-regulators and pathogenic factors. Overall, the etiology and potential pathology of c-aAb are still undefined. To further characterize the sero-prevalence, predictors and consequences of high c-aAb levels, we performed the largest population-based study of c-aAb to date, using participants and epidemiological data from the Danish Blood Donor Study. Using a validated bead-based multiplex assay we assessed plasma levels of IL-1α, IL-6, IL-10, IFNα and GM-CSF-specific c-aAb in 8,972 healthy blood donors. Trace levels of at least one of the investigated c-aAb could be measured in 86% of the participants. The presence of high levels of potentially inhibitory c-aAb was generally associated with increasing age and male or female sex, depending on the c-aAb in question. A negative correlation between high levels of IL-6-specific c-aAb and plasma levels of C-reactive protein was observed, indicating cytokine-neutralizing levels of c-aAb in healthy blood donors. There was no substantial correlation between high levels of the five individual c-aAb investigated in this study. These data suggest that autoimmunity against endogenous cytokines is a relatively common phenomenon in healthy individuals, and that predictive factors for high, potentially neutralizing c-aAb levels vary depending on the cytokine in question, and may differ from predictors of general c-aAb presence.

## Introduction

Cytokines play key roles in inflammation and cell biology [1]. They are produced and act in femto- to nano-molar concentrations, and are extremely bio-potent compared to endocrine hormones [2]. Systemically, cytokines are normally present at very low concentrations. This is in contrast to inflamed or immunologically active tissues where cytokine concentrations may reach nanomolar concentrations [3]. Such conditions impose strict requirements for the induction of immunological tolerance to endogenous cytokines, which has been shown to be governed by the autoimmune regulator protein in the thymus [4]. In general, signaling cells produce a refined mix of different cytokines, some of which exert overlapping functions [5]. This redundancy in the cytokine network implies that the functional lack of one cytokine or cytokine receptor rarely leads to overt pathology. However, due to their central roles in inflammation and immunity, certain dysregulations of the cytokine network may predispose individuals to infections or autoimmune disorders. One example of a pathological cytokine deficiency is pulmonary alveolar proteinosis (PAP), a rare disorder involving respiratory restriction and increased susceptibility to infection [6]. PAP originates from dysregulated macrophage development resulting in impaired mucus clearance, with macrophage development relying on the cytokine granulocyte macrophage colony-stimulating factor (GM-CSF)[7]. Functional inhibition of cytokines may be caused by cytokine-specific autoantibodies (c-aAb), and a strong link between PAP and neutralizing levels of GM-CSF-specific c-aAb has been established [7–9]. Several other c-aAb have been linked to lacunar immune deficiencies [10–15], and deficiency of the autoimmune regulator protein is irrevocably linked to the presence of neutralizing Interferon (IFN) -specific c-aAb. This is observed in patients suffering from autoimmune polyendocrinopathy candidiasis ectodermal dystrophy (APECED), a disorder causing highly variable phenotypes including hypoparathyroidism, adrenal dysfunction and increased susceptibility to *Candida* infections [16–18]. *Candida* risk for APECED patients has been specifically correlated to IL-17 and -22-specific c-aAb [19].

Over the last decade, multiple studies have reported high avidities of IgG c-aAb in various patient cohorts, and relatively high levels of high avidity c-aAb in pharmaceutically prepared pools of plasma-derived human polyclonal IgG [3, 20–24]. Together, these findings suggest that both B-cell and T-cell tolerance to endogenous cytokines is vulnerable, and that breaking this tolerance may occur in both diseased and healthy individuals [25–27]. C-aAb may also be induced in patients in response to cytokine therapy, with such antibodies having the potential to interfere with treatment [28–30].

Although c-aAb therefore appear to be a potential risk factor for a range of disorders, including immune deficiency, their presence in apparently healthy individuals gives rise to a mixed picture of their exact clinical significance. Furthermore, c-aAb against pro-inflammatory cytokines such as IL-1α and IL-17 have been observed to correlate with decreased disease severity in arthritis patients [31–34], and have been speculated to act as a protective mechanism. C-aAb in general have also been proposed to act as endogenous immunoregulators [35]. Whether c-aAb represents a regulatory mechanism or a dysfunction of immunologic tolerance is an open question, but it is clear that c-aAb mediated cytokine neutralization might have decisive effects on health.

In order to further understand the role of c-aAb, we wanted to investigate the prevalence and significance of high levels of naturally occurring c-aAb in a healthy population, to identify epidemiological predictors, and create a reference work for future studies. For this purpose we utilized participants in the Danish Blood Donor Study (DBDS), a well-characterized population with pre-existing epidemiological and biomarker data. Thus, using blood donors as representatives of a healthy population we were able to screen 8,972 individuals for the presence of five c-aAb which had been previously characterized by our group [3, 24, 25, 28, 30, 36–41]. We found c-aAb to be a common phenomenon. Age, sex and smoking status correlated with the presence of detectable c-aAb levels, and age and sex correlated with increased susceptibility to high levels of individual c-aAb. A functional effect of elevated levels of interleukin-6 (IL-6)-specific c-aAb was suggested by an association to lower blood C-reactive protein (CRP) levels.

## Materials and methods

### Coupling of cytokine to microspheres

Cytokine-coupling was performed as previously described [41]. Briefly, MagPlex Microspheres (Luminex Corp., Austin, Texas) were activated using 100 mM 6.2 pH NaH_2_PO_4_ (Sigma-Aldrich, St. Louis, Missouri), containing 2.5 mg/ml N-hydroxysulfosuccinimide (Sigma-Aldrich) and N-(3 dimethylaminopropyl)-N′-ethylcarbodiimide hydrochloride (Sigma-Aldrich). Beads were washed in PBS (pH 7.4) and incubated with PBS-dissolved cytokine (4 μg/1.25 * 10^6^ beads) for 2 hours at room temperature, followed by blocking for 30 minutes using PBS containing 2% BSA(Sigma-Aldrich), 0.5% (v/v) Tween 20 and 0.05% (w/v) Sodium Azide. Beads were finally washed and re-suspended in blocking buffer and stored at 4°C in the dark until use. Recombinant human carrier-free IL-1α, IL-6, IL-10, IFNα (isoform IFNA2a) and GM-CSF were purchased from R&D systems (Abingdom, United Kingdom).

### Anti-cytokine autoantibody multiplex assay

The indirect, pseudo-quantitative serological assays were performed as previously described [41], with minor modifications. In brief, plasma was mixed with assay buffer and cytokine-conjugated MagPlex beads for a final 10-fold plasma dilution. The mixture was incubated for 1 hour at room temperature with gentle shaking. Beads received 3 washes in assay buffer, and 2.5 μl secondary polyclonal goat F (ab’)_2_ anti-human IgG PE-conjugated antibody (Catalog #H10104, Thermo Fisher Scientific, Waltham, Massachusetts) in 100 μl assay buffer dilution was added to each well. Following 30 minutes of incubation, the beads received another 3 washes and assays were stored overnight at 4°C in assay buffer. The following day assays were analyzed using the Luminex 100 system (Luminex Corp.) and StarStation software (Luminex Corp.). Median Fluorescence Intensity (MFI) was determined for the MagPlex beads of each sample. MFI indirectly reflects the amount of free c-aAb cytokine binding sites in a sample, and was chosen as a pseudo-quantitative measure of sample c-aAb concentration. Prior studies have confirmed the cytokine specificity of measured c-aAb MFI signals, and previously characterized c-aAb negative plasma was used as a negative control. As a positive control we used a pool of 5 plasma samples with high and c-aAb-specific MFI values, at an 8-fold final pool dilution. Participants with MFI values greater than the negative control + 4 standard deviations (SD) were classified as having a positive c-aAb signal, as these cutoffs corresponded to previously observed lower limits of specific c-aAb signals. The average MFI values (with SD) of the negative control were 127 (± 27), 75 (± 15), 50 (± 9), 44 (± 9) and 99 (± 27) for IL-1α, IL-6, IL-10, IFNα and GM-CSF-specific c-aAb respectively, and the average MFI’s for the positive control were 8,194 (± 733), 18,704 (± 1,410), 1,797 (± 235), 4,073 (± 522) and 7,085 (± 729). Samples were run in monoplicates.

For the IgG-pool experiments we utilized Privigen^®^ from CSL Behring (Bern, Switzerland), corresponding to approximately 20,000 donors each. IgG pools for this study were based on Danish plasma, as part of a tender between the Danish health care system and CSL Behring (http://www.cslbehring.dk/fra-dansk-donor-til-dansk-patient). Privigen^®^ was serially diluted in assay buffer and incubated with beads as above. Signal specificity was tested by 1 hour of pre-incubation of serially diluted Privigen^®^ with 100 nM of each cytokine, prior to mixing with cytokine-conjugated beads.

### Participants

Samples were obtained from the first 20,000 participating blood donors from DBDS [42–45], a prospective study which consists of more than 110,000 participants as of April 2017 (www.dbds.dk). Participating blood donors completed a questionnaire (Images in S1 file), granted permission for routine blood samples to be used for research, and represent healthy individuals within an age range of 18–67 years. Participant body mass index (BMI) was calculated as self-reported weight (kg) divided by self-reported height (m) squared. BMI values were discarded in the case of unrealistic anthropometric outliers. Obesity was defined as having BMI > 30. Smokers were defined as “active use of tobacco” at the time of inclusion, with “active use of tobacco” itself defined as having given the first or second answer to question 17 (“do you smoke”), and skipping to question 20 as instructed (Images in S1 file). CRP levels were measured in plasma samples taken upon inclusion of the participants using the Vitros^®^ 5600 integrated system (Ortho Clinical Diagnostic, NJ, USA). Plasmas were stored at -20°C prior to analysis and thawed once prior to CRP measurement, and once more prior to c-aAb measurement. For 892 of the included participants, information on smoking status, weight/height, or CRP level was missing.

### Ethical considerations

Upon enrollment into the study, participants provided written and oral informed consent. The described study investigated normally occurring c-aAb in healthy individuals. No definitive causative association between c-aAb in healthy individuals and disease risk has been established. This study was approved in the Danish ethical committee system under the code numbers 01-110/98, H-15000761 and M-20090237. The biobank and research database was approved by the Danish Data Protection Agency (2007-58-0015). The study was conducted according to the principles of the Helsinki Declaration.

### Statistical analysis

Chi-squared tests were used to test for associations between the presence of positive or high levels of the five investigated c-aAb. Positive levels of c-aAb for individual participants were defined as MFI values above the previously listed negative background + 4 SD, and high c-aAb levels were defined as MFI above the 99^th^ MFI percentile of the population.

Logistic regression analyses were used for identification of predictors of high levels of c-aAb. First we performed a series of univariate analyses using age (expressed in decades), sex, obesity (BMI>30), current smoking habits and CRP as independent variables, and the dichotomous outcome of having high levels of c-aAb or not as the dependent variable. CRP was log-transformed in all analyses involving continuous CRP data, in order to approximate a normal distribution. Subsequently, we performed multivariate logistic regression analysis using all the aforementioned independent variables. Logistic regression analyses were also applied to evaluate predictors of positive c-aAb MFI values, using the independent variables listed above.

Multivariate linear and logistic regression analyses were performed to assess c-aAb as predictors for CRP levels. Linear regression analyses were based on log-transformed CRP as the dependent variable, and logistic regression analyses were based on a dichotomous variable called “CRP-low” as the dependent variable. The CRP detection limit of the assay was 0.1 mg/L, and participants with CRP < 0.1 mg/L were classified as “CRP-low”. The regression analyses included high levels of c-aAb, age, sex, obesity and smoking status as independent variables.

T-tests were performed to test for differences between men and women for normally distributed variables (age, BMI and log-transformed CRP), as well as the relation of c-aAb MFI values to age, sex obesity and smoking. Chi-squared tests were done for the correlation of dichotomous variables (sex, smoking status and “CRP-low”). The general distribution of c-aAb MFI values as stratified by age (above/below the mean age of the population), sex, obesity and smoking status, were analyzed by Wilcoxon rank-sum tests.

Statistical analyses were made using the STATA software (STATA/IC 14 for PC, StataCorp, College Station, TX). *P* values below 0.05 were considered statistically significant.

## Results

### Characteristics of the cohort

The study population comprised 8,972 unique healthy blood donors, who were enrolled in the DBDS cohort between March 1st and December 31st, 2010. The basic characteristics of the study population are summarized in Table 1. Data shown are based on participants with available questionnaire data relevant to the respective parameters. Men were significantly older than women, had a higher BMI, and lower levels of CRP (*p* < 0.001).

**Table 1:** Characteristics of the study population (n = 8,972).

	Men (n = 4,680)	Women (n = 4,292)	Total (n = 8,972)	*p* value, men vs women*
Age (years)**	41 (± 12.2)	38.7 (± 12.4)	39.9 (± 12.4)	< 0.001
**BMI (kg/m**^**2**^)**	25.6 (± 3.5)	24.5 (± 4.1)	25.1 (± 3.9)	< 0.001
Non-obese: BMI<30***	89.5% (n = 3,887)	89.7% (n = 3,633)	89.6% (n = 7,520)	0.810
Obesity: BMI≥30***	10.5% (n = 455)	10.3% (n = 418)	10.4% (n = 873)	
**Smokers** ***	16.4% (n = 709)	18% (n = 722)	17.2% (n = 1,431)	0.055
Non-smokers***	83.6% (n = 3,610)	82% (n = 3,289)	82.8% (n = 6,899)	
CRP (mg/L)****	0.4 (0.1;1.2)	0.7 (0.2;2.0)	0.6 (0.2;1.5)	< 0.001
Undetectable:<0.1 mg/L***	23.8% (n = 1,098)	18.4% (n = 775)	21.2% (n = 1,873)	< 0.001
Detectable:>0.1 mg/L***	76.2% (n = 3,513)	81.6% (n = 3,437)	78.8% (n = 6,950)	

*Statistical significant difference tested through t-tests for continuous variables (age, BMI and log10 transformed CRP), and chi-squared tests for dichotomous variables (sex, obesity, smoking, detectable CRP levels)

** Data presented as mean ± SD

*** Data presented as percentage of group

**** Data presented as median with interquartile range

### Levels of c-aAb in plasma from healthy blood donors

**Fig 1:**
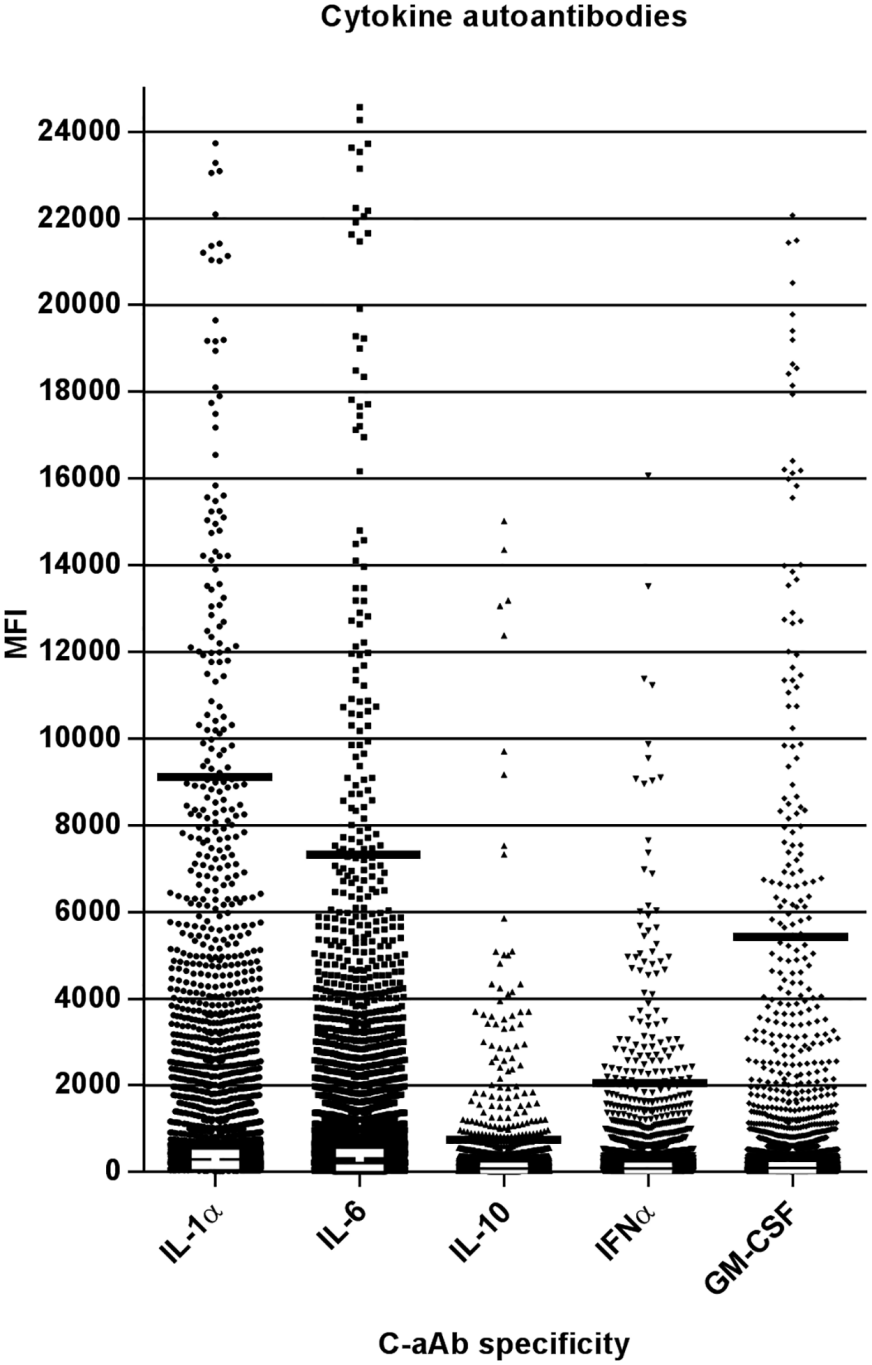
Distribution of MFI for five c-aAb in healthy blood donors. Plasma samples from 8,972 healthy participants were diluted 10-fold and incubated with cytokine-conjugated MagPlex beads. High levels of c-aAb MFI signals were defined as the 99^th^ MFI percentile (black lines), as described in materials and methods. White lines indicate median with interquartile range. One screening was performed per sample.

To assess the distribution of c-aAb plasma levels we performed a multiplex screening of IL-1α, IL-6, IL-10, IFNα and GM-CSF-specific c-aAb. Participants having MFI values above the negative control mean + 4 SD were considered positive for c-aAb. IL-6-specific c-aAb was the most prevalent c-aAb, being detectable in 65% of all participants, and GM-CSF-specific c-aAb appeared to be the rarest with a prevalence of 10%. Overall, 85.8% of the participants were positive for at least one of the five investigated c-aAb (Fig 1 and Table 2).

**Table 2:** Distribution of c-aAb in the study population (n = 8,972).

C-aAb specificity	MFI cutoff for c-aAb”positive”**	Frequency c-aAb”positive”*	MFI cutoff for c-aAb”high”**	Frequency c-aAb “High”**
**IL-6**	135	65% (n = 5,835)	7.461	1% (n = 90)
**IL-1α**	235	48.5% (n = 4,349)	9,001	1% (n = 90)
**IL-10**	86	35.5% (n = 3,186)	1,051	1% (n = 90)
**IFNα**	80	34% (n = 3,048)	2,129	1% (n = 90)
**GM-CSF**	207	10% (n = 894)	5,487	1% (n = 90)
**Any of the above**	See above	85.8% (n = 7,562)	See above	4.9% (n = 441)

* MFI > mean negative control + 4 SD

** MFI > 99^th^ MFI percentile

### Levels of c-aAb in healthy Danish blood donor-derived IgG pools

**Fig 2:**
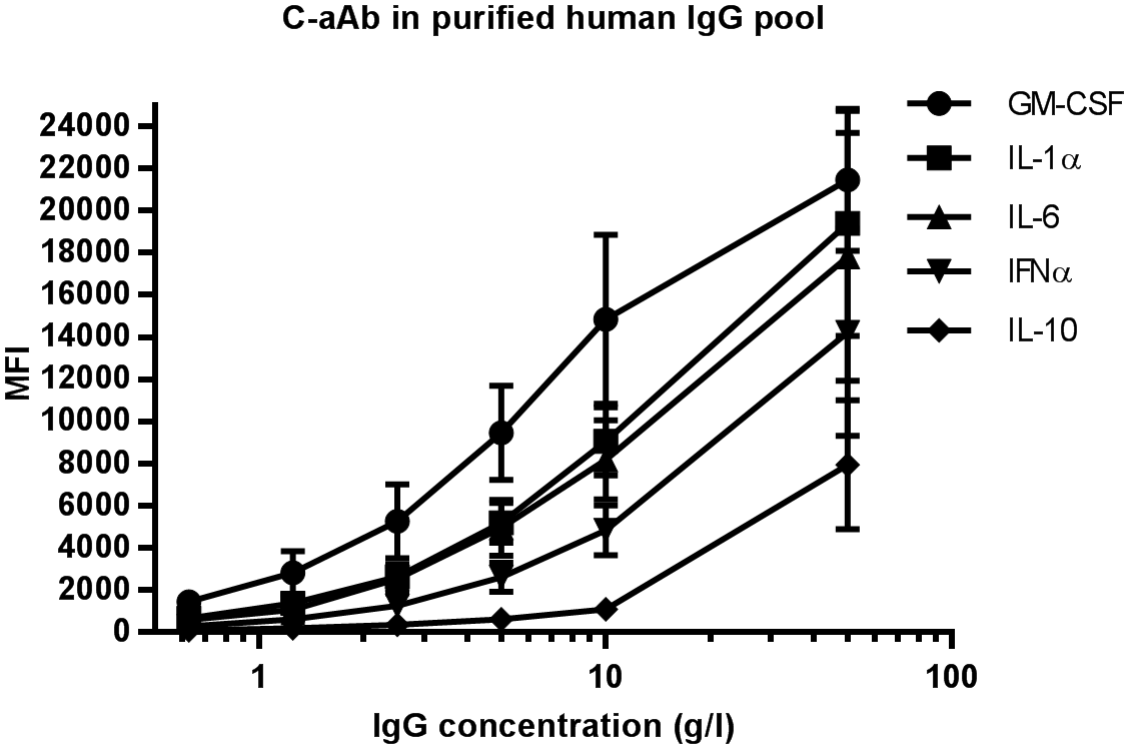
IgG pool c-aAb dilution. A pool of IgG derived from Danish blood donors (Privigen^®^) was serially diluted in assay buffer followed by incubation with cytokine-conjugated MagPlex beads and analysis on a Luminex 100 platform. The IgG pool c-aAb levels were analyzed at a final dilution range of 0.625 to 50 mg/ml. The data shown represent averages with SD for experiments with 3 individual lots of Privigen^©^.

To further assess the prevalence of c-aAb, we screened for IL-1α, IL-6, IL-10, IFNα and GM-CSF-specific c-aAb in commercial IgG pools derived from approximately 20,000 healthy Danish blood donors (Privigen^®^). The assay detected the presence of all five c-aAb in three separate IgG pools (Fig 2). In order to confirm the signal specificity, IgG pool dilutions were pre-incubated with 100 nM of free cytokines prior to mixing with cytokine-conjugated beads. We observed high levels of signal reduction (41–83%) for IL-1α, IL-6, IFNα and GM-CSF-specific c-aAb in cytokine pre-treated samples and a moderate reduction (8–43%) for IL-10-specific c-aAb (Figure in S2 file).

### Correlation of individual c-aAb levels

In order to investigate the relationship between different c-aAb, we performed chi-squared tests for correlation of the presence of positive MFI values for the five c-aAb. MFI levels of all five c-aAb were positively correlated with each other (p < 0.001, Table 3). We defined a subset of donors with c-aAb MFI values above the 99^th^ percentile to have high levels of c-aAb (Fig 1). We observed no significant association between high levels of the different c-aAb, with the exception of GM-CSF and IL-1α-specific c-aAb (3/8,972 participants, *p* = 0.026, Table 3).

**Table 3:** Correlation of c-aAb levels.

			**IL-1α c-aAb**	**IL-6 c-aAb**	**IL-10 c-aAb**	**IFNα c-aAb**	**GM-CSF c-aAb**
**IL-1α c-aAb**	C-aAb MFI positive*	**Test statistic**	-	644	209	711	1,100
		***p* value**	-	<0.001	<0.001	<0.001	<0.001
	High c-aAb MFI**	**Test statistic**	-	0.011	0.011	0.011	4.971
		***p value***	-	0.918	0.918	0.918	0.026
**IL-6 c-aAb**	C-aAb MFI positive*	**Test statistic**	644	-	103	500	604
		***p value***	<0.001	-	<0.001	<0.001	<0.001
	High c-aAb MFI**	**Test statistic**	0.011	-	0.921	1.361	0.921
		***p value***	0.918	-	0.337	0.243	0.337
**IL-10 c-aAb**	C-aAb MFI positive*	**Test statistic**	209	103	-	180	229
		***p* value**	<0.001	<0.001	-	<0.001	<0.001
	High c-aAb MFI**	**Test statistic**	0.011	0.921	-	0.011	0.921
		***p* value**	0.918	0.337	-	0.918	0.337
**IFNα c-aAb**	C-aAb MFI positive*	**Test statistic**	711	500	180	-	1,000
		***p* value**	<0.001	<0.001	<0.001	-	<0.001
	High c-aAb MFI**	**Test statistic**	0.011	1.361	0.011	-	0.011
		***p* value**	0.918	0.243	0.918	-	0.918
**GM-CSF c-aAb**	C-aAb MFI positive*	**Test statistic**	1,100	604	229	1,000	-
		***p* value**	<0.001	<0.001	<0.001	<0.001	-
	High c-aAb MFI**	**Test statistic**	4.971	0.921	0.921	0.011	-
		***p* value**	0.026	0.337	0.337	0.918	-

* The presence of c- aAb positivity were defined as MFI values above the negative control + 4 SD, and were correlated using chi-squared tests

** High levels of c-aAb were defined as MFI values above the 99^th^ percentile, and were correlated using chi-squared tests

### Age, sex, obesity and smoking as predictors of c-aAb levels

Through Wilcoxon rank-sum tests, we assessed the overall distribution of c-aAb MFI values in relation to age, sex, obesity and smoking habits. Negative correlations of MFI medians to age, female sex, and smoking status were observed (figure in S3 File). Using univariate and multivariate logistic regression analyses, we then investigated these epidemiological parameters as potential predictive factors for having high levels of the individual c-aAb. In the initial univariate analyses, these variables again expressed *p* values of ≤ 0.1 for at least one of the investigated c-aAb, and were thus included in the subsequent multivariate logistic regression analyses (Table 4). In the following, we will focus on results from these multivariate logistic regression analyses, in addition to further analyses investigating the combined epidemiological variables as predictors of detectable c-aAb MFI signals (figure in S4 File).

**Table 4:** Univariate and multivariate analysis of predictors of high levels of c-aAb*.

			Age (decades)	Sex (women = 1)	Obesity (obese = 1)	Smoking (smoker = 1)	CRP (log mg/L)
**High levels of IL-1α c-aAb**	Univariate analysis**	**OR (95% CI)**	1.67 (1.40–1.98)	0.39 (0.25–0.63)	1.71 (0.96–3.05)	0.51 (0.23–1.10)	1.09 (0.95–1.25)
		***p* value**	<0.001	<0.001	0.069	0.079	0.240
	**Multivariate analysis** ***	**OR (95% CI)**	1.60 (1.34–1.91)	0.44 (0.27–0.72)	1.29(0.79–2.40)	0.50 (0.23–1.09)	1.09 (0.92–1.28)
		***p* value**	<0.001	0.001	0.223	0.083	0.569
**High levels of IL-6 c-aAb**	Univariate analysis**	**OR (95% CI)**	1.24 (1.05–1.47)	1.37 (0.90–2.08)	1.42 (0.77–2.63)	1.19 (0.67–2.12)	0.82 (0.71–0.94)
		***p* value**	0.010	0.142	0.262	0.560	0.006
	**Multivariate analysis** ***	**OR (95% CI)**	1.28 (1.08–1.52)	1.53 (0.99–2.38)	1.56 (0.78–3.14)	1.22(0.69–2.19)	0.65 (0.76–0.88)
		***p* value**	0.002	0.055	0.204	0.487	0.000
**High levels of IL-10 c-aAb**	Univariate analysis**	**OR (95% CI)**	1.31 (1.12–1.56)	0.66 (0.43–1.01)	0.54 (0.22–1.33)	1.12 (0.63–1.99)	1.09 (0.95–1.26)
		***p* value**	0.001	0.057	0.177	0.701	0.214
	**Multivariate analysis** ***	**OR (95% CI)**	1.32 (1.12–1.57)	0.67 (0.43–1.05)	0.48 (0.19–1.20)	0.98 (0.56–1.85)	1.14 (0.98–1.33)
		***p* value**	0.001	0.062	0.073	0.950	0.786
**High levels of IFNα c-aAb**	Univariate analysis**	**OR (95% CI)**	0.98 (0.83–1.16)	1.43 (0.94–2.18)	0.74 (0.34–1.61)	0.59 (0.29–1.23)	1.04 (0.91–1.20)
		***p* value**	0.830	0.094	0.451	0.162	0.545
	**Multivariate analysis** ***	**OR (95% CI)**	1.01 (0.84–1.20)	1.38 (0.89–2.14)	0.72 (0.31–1.60)	0.57 (0.28–1.28)	1.06 (0.91–1.23)
		***p* value**	0.921	0.156	0.418	0.130	0.473
**High levels of GM-CSF c-aAb**	Univariate analysis**	**OR (95% CI)**	1.23 (1.05–1.46)	1.09 (0.72–1.65)	1.17 (0.60–2.27)	0.70 (0.35–1.40)	0.99 (0.86–1.13)
		***p* value**	0.013	0.680	0.650	0.308	0.855
	**Multivariate analysis** ***	**OR (95% CI)**	1.22 (1.05–1.48)	1.24 (0.79–1.93)	0.90 (0.42–1.92)	0.61 (0.30–1.29)	0.95 (0.88–1.17)
		***p* value**	0.017	0.353	0.792	0.197	0.567

* High levels of c-aAb defined as MFI values above the 99^th^ percentile

** Univariate logistic regression analyses with high c-aAb levels as the dependent variable, and age, sex, obesity, smoking and CRP as alternate independent variables. Obesity was defined as having BMI>30, smoking was defined as being an active consumer of tobacco at the time of sampling, and CRP (mg/l) was log-transformed prior to analysis. Age was expressed in decades.

*** Multivariate logistic regression analyses with high c-aAb levels as the dependent variable, and age, sex, obesity, smoking and CRP (defined as above)as the independent variables.

#### IL-1α-specific c-aAb

**Fig 3:**
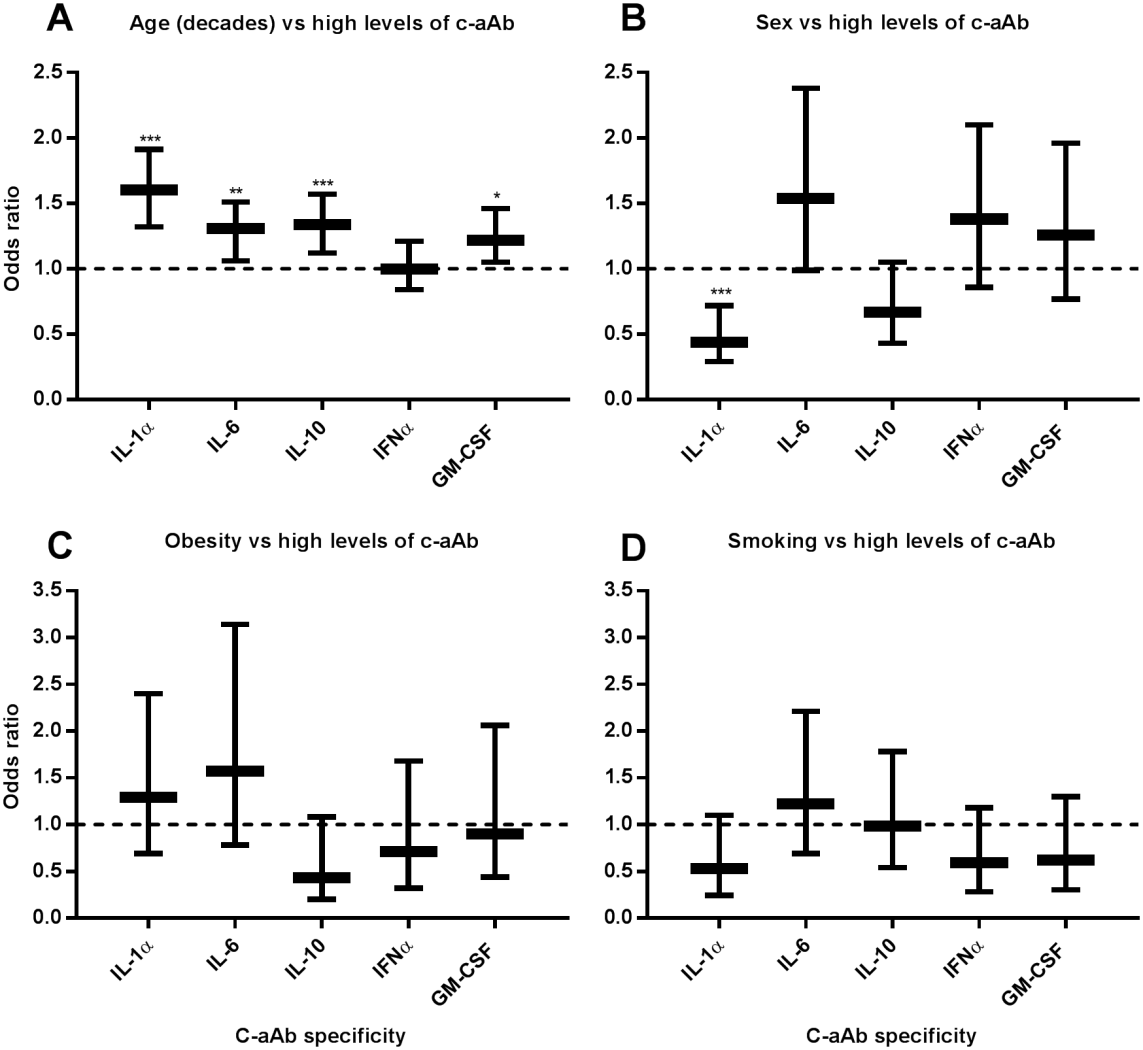
Correlation between c-aAb positivity and epidemiological parameters. Multivariate logistic regression analysis was used to investigate the predictors of high levels of individual c-aAb. Age, sex, obesity, smoking and CRP were used as independent variables and high levels of c-aAb as the dependent variable. For dichotomous variables high levels of c-aAb, female sex, active smoking and obesity were defined as “1”. Data are presented as OR with 95% confidence interval for age, sex, obesity, and smoking (panels A-D) as predictors of high levels of c-aAb. * denotes a *p* value of < 0.05, **p < 0.01, and *** *P* ≤ 0.001.

Increased age was associated with high levels of IL-1α-specific c-aAb (Odds Ratio (OR) = 1.60, 95% Confidence Interval (CI) = 1.34–1.91, *p* ˂ 0.001, Fig 3A, Table 4). Women were significantly less likely to express IL-1α-specific c-aAb at high levels (OR = 0.44, 95% CI = 0.27–0.72, *p* = 0.001, Fig 3B, Table 4). In logistic regressions using positive c-aAb MFI levels as the dependent variable, a significant positive correlation to male sex was again observed (*p* ˂0.001, Figure B in S4 File), along with a negative association with smoking (*p* = 0.053, Figure D in S4 File). A negative correlation to age was also observed for positive c-aAb levels (*p* ˂0.001, Figure A in S4 File).

#### IL-6-specific c-aAb

Increased age was associated with highly elevated IL-6- specific c-aAb (OR = 1.28, CI = 1.08–1.52, *p* = 0.002, Fig 3A, Table 4), yet negatively associated with the presence of detectable c-aAb MFI (*p* < 0.001, Figure A in S4 File). High levels of IL-6-specific c-aAb tended to be positively associated with female sex, (p = 0.055, Fig 3B, Table 4).

#### IL-10-specific c-aAb

High levels of IL-10-specific c-aAb were associated with increased age (OR = 1.32, 95% CI = 1.12–1.62, *p* = 0.001, Fig 3A, Table 4). Women tended to be less likely to present IL-10-specific c-aAb at high levels (OR = 0.67, 95% CI = 0.43–1.05, *p* = 0.062, Fig 3B, Table 4), as well as positive levels of IL-10 c-aAb overall (*p* < 0.001, Figure B in S4 File). Positive levels of IL-6 c-aAb were also negatively correlated with smoking (*p* < 0.001, Figure D in S4 File)

#### IFNα-specific c-aAb

None of the investigated epidemiological factors were observed to be predictors of high levels of IFNα-specific c-aAb in uni- or multivariate logistic regression analyses (Fig 3, Table 4). Positive IFNα specific c-aAb levels were negatively correlated with age (*p* < 0.001, Figure A in S4 File), female sex (*p* = 0.046, Figure A in S4 File), and smoking status (*p* < 0.001, Figure D in S4 File).

#### GM-CSF-specific c-aAb

Increased age was associated with GM-CSF-specific c-aAb at high levels (OR = 1.22, 95% CI = 1.05–1.48, *p* = 0.017, Fig 3A, Table 4). Female sex and smoking status were negatively correlated to positive c-aAb levels (*p* < 0.001, Figures B and D in S4 File).

### High levels of IL-6-specific c-aAb as a predictor of low CRP levels

**Fig 4:**
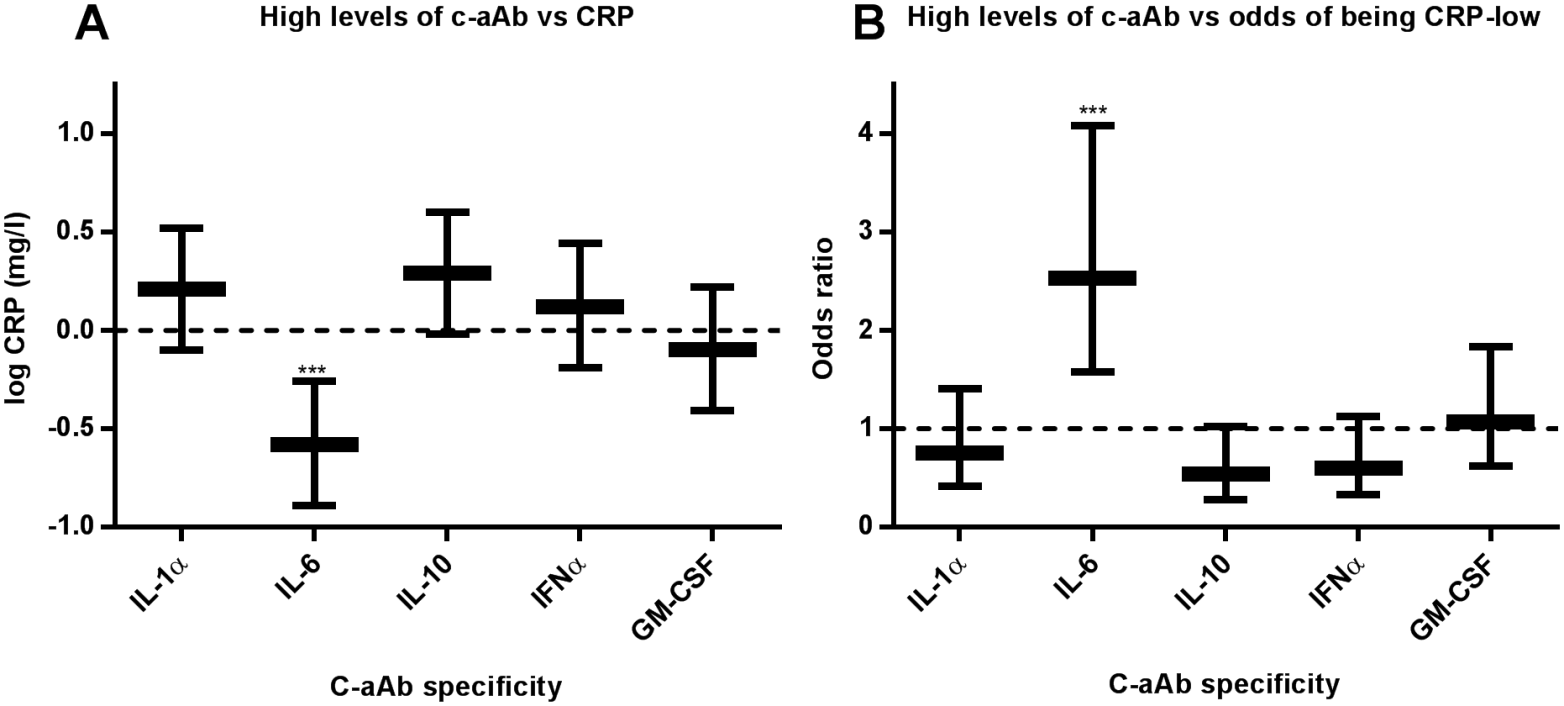
C-aAb as a predictor of CRP. A: Log-transformed CRP was used as the dependent variable in a series of linear regression analyses, with sex, age, obesity, active smoking and high levels of c-aAb as independent variables. High levels of c-aAb were defined as MFI values above the 99^th^ percentile. Data are presented as regression coefficients with 95% confidence intervals. B: Participant status of being CRP-low (CRP < 0.1 mg/L) was used as the binary dependent variable in a series of logistic regression analyses, with sex, age, obesity, active smoking and high levels of c-aAb as independent variables. Data are presented as OR in favor of being CRP-low, with 95% confidence intervals. ***denotes a *p* value of ≤ 0.001.

In order to investigate c-aAb mediated cytokine-neutralization in healthy individuals, we evaluated c-aAb as predictors of the levels of the inflammatory marker CRP. Using log-transformed CRP as the dependent variable we performed multivariate linear regression analyses, and observed a significant negative correlation between high levels of IL-6-specific c-aAb and CRP (p < 0.001, Fig 4A). We then investigated high levels of c-aAb as predictors of having undetectable CRP levels (< 0.1 mg/L), which we defined as “CRP-low” (Table 1). Through logistic regression analyses we again observed a significant association of high levels of IL-6-specific c-aAb and “CRP-low” status (OR = 2.53, 95% CI = 1.57–4.08, *p* < 0.001), and no significant association between CRP and other c-aAb (Fig 4B).

## Discussion

In this study 86% of a normal healthy blood donor population expressed detectable levels of at least one of five c-aAb, demonstrating that loss of immunological tolerance against certain cytokines is a common feature in humans. This finding was substantiated by the relatively high c-aAb levels in pharmaceutically prepared IgG pools based on plasma from 20,000 Danish blood donors. IL-1α and IL-6-specific c-aAb appeared to be the most common, with a sero-prevalence of 48.5 and 65%, respectively. In prior studies the estimated prevalence of IL-1α-specific c-aAb has ranged from 5 to 40% in healthy individuals, based on a variety of criteria for being c-aAb positive [46–48], indicating a necessity for high-powered c-aAb screenings of healthy individuals using validated assays. IL-1α-specific c-aAb have generally appeared to be more common than other c-aAb, in line with our results [49]. GM-CSF-specific c-aAb were the most rare and yet, as previously observed, the GM-CSF-specific c-aAb signal was the most predominant in the IgG pools [24]. This supports the notion that the prevalence and levels of c-aAb may vary considerably depending on the cytokine in question, and we speculate that a few extremely positive donors may dominate the binding capacity of a large IgG pool. The lower levels of detectable IL-10 and IFNα-specific c-aAb are in line with the existing literature [24, 50], but could also be due to a lower assay sensitivity based on variable efficiency of cytokine-coupling to MagPlex beads. For this reason, we defined “high levels” of c-aAb as the 99^th^ MFI percentile for the respective c-aAb.

Though there was a significant positive correlation between positive levels of the various c-aAb, this correlation did not extend to high c-aAb levels, with the exception of a slight positive correlation between IL-1α and GM-CSF-specific c-aAb. Younger age was a significant predictor of detectable c-aAb for most of the antibodies investigated, yet the most common predictor of high levels of c-aAb was age, which had a significant positive association to all c-aAb except for the IFNα-specific. With a maximum age of 67 years, the number of elderly individuals in our population was limited, and it will therefore be important to further explore the c-aAb prevalence in cohorts of older individuals, as well as their potential relevance in certain age-related conditions. This may be particularly relevant for IL-1α and IL-10-specific c-aAb, as reports have indicated a decline in the systemic levels of these cytokines with age [51, 52]. If present, c-aAb could thus further impair an already reduced cytokine response.

We confirmed a previously established association of high IL-1α-specific c-aAb levels with male sex [48]. Additionally, men tended to have high levels of IL-10-specific c-aAb, and increased likelihood of detectable IL-1α, IL-10, IFNα and GM-CSF specific c-aAb. Uniquely among the investigated antibodies, women tended to have high levels of IL-6 c-aAb. Obesity is increasingly understood to influence inflammation and autoimmunity [44, 53], and previous findings have implicated IL-6-specific c-aAb in type 2 diabetes and obesity [37], yet we did not find obesity to be a predictive factor for high or detectable levels of c-aAb. Likewise, smoking has been established as a potential inducer of autoimmunity [54], yet a general negative correlation of smoking was found to the odds of having detectable c-aAb levels, and no significant association was found to high levels of c-aAb. Thus, it appears that the etiologies of highly elevated c-aAb levels vary from those of generally detectable c-aAb levels. In addition, apart from the almost uniformly observed positive correlation with age, the etiologies of highly elevated c-aAb levels seem to vary according to the c-aAb in question, with male sex expressing a positive tendency for high IL-1α and IL-10 c-aAb, and female sex an association to high levels of IL-6 c-aAb. This variable etiology is supported by the limited overlap between high levels of the different c-aAb in individual donors.

We observed a significant negative correlation between high levels of IL-6-specific c-aAb and plasma CRP levels, and high-levels of IL-6 c-aAb correlated with undetectable levels of CRP. As CRP is an inflammatory marker induced by IL-6 [55], and since this correlation was unique to IL-6-specific c-aAb, these data suggest the presence of cytokine inhibitory levels of IL-6-specific c-aAb. This finding is in concordance with previous experimental observations [11–13, 39, 40]. Apparently the correlation was not affected by smoking status, despite smoking being known to affect CRP levels [44, 56]. The exact scale of c-aAb mediated functional cytokine suppression in healthy plasmas will need to be determined in future in vitro bioassays. It is worth noting that the levels of IL-6-specific c-aAb in the IgG pools were well below the potentially IL-6 inhibiting threshold when diluted to plasma-equivalent antibody concentrations. This suggests that clinical use of IgG pools at normal concentrations does not cause substantial IL-6 neutralization. The relationship between IL-6-specific c-aAb and CRP raises the question of whether individuals with high levels of other c-aAb may express currently neglected signs of functional cytokine suppression. The presence of high levels of c-aAb in apparently asymptomatic individuals, however, supports the notion that cytokine redundancy may effectively compensate for single-cytokine deficiencies. Blood donors may thus be healthy carriers of inhibitory levels of c-aAb, which may in turn be passed on to patients via blood transfusion.

Further studies are needed to determine the exact influence of c-aAb in apparently asymptomatic individuals, as well as the effect of duration of exposure to high c-aAb levels on health outcome; It is possible that the observed correlation between c-aAb and CRP or previously established links to diseases such as PAP are caused by long-lasting c-aAb responses, and that temporarily elevated levels of c-aAb represent endogenous regulation, which does not lead to such effects.

### Strengths and limitations of the study

The study utilized an assay that has been extensively validated using samples with known c-aAb activity, as determined by radio-immunoassays. The assay has also been tested for cross-cytokine reactivity. As the MFI signal is determined by the number of free antigen binding sites, it is possible that a part of the MFI signal represents immune complexes with free antigen binding sites. Alternately, immune complexes without free binding sites may mask c-aAb MFI signals. The measurements of IL-10 c-aAb and data relating to displacement of the MFI values of IgG pools indicate that the sensitivity of the assay may be low for IL-10-specific c-aAb. IL-10 is a dimer, and it is possible that the coupling of IL-10 on MagPlex beads denature IL-10 in such a way that certain epitopes are lost. It is an attention point that we intend to examine more closely.

The nature of our population allowed us to exclude disease and medical treatment as factors influencing c-aAb levels, but this could also be seen as a limitation of our study. Blood donors are healthier than the general population, and the use of this selected population may have caused an underestimation of the impact of c-aAb on health. A long-lasting c-aAb response could cause exacerbated effects of cytokine neutralization, eventually excluding the individual from donating blood and thus participating in DBDS. Furthermore, anthropometric parameters such as weight and height were self-reported.

## Conclusion

We observed detectable levels of IL-1α-, IL-6-, IL-10-, IFNα- and GM-CSF-specific c-aAb in the plasma of 86% of Danish blood donors, with young age, male sex and non-smoking status as the most common predictors of increased likelihood of detectable c-aAb. The prevalence of c-aAb varied depending on c-aAb specificity, with IL-1α and IL-6-specific c-aAb being the most prevalent. The epidemiological predictors of high c-aAb levels included advancing age and variable sex, depending on the c-aAb in question. Though a general positive association between different c-aAb MFI levels was observed, there was only minor overlap between highly elevated levels of the five investigated c-aAb, suggesting different etiologies depending on the c-aAb in question. Our data confirmed that high levels of IL-6-specific c-aAb have a significant negative association with CRP plasma levels, and thus suggest that healthy blood donors may harbor c-aAb at cytokine-inhibiting levels. Neutralizing levels of c-aAb in apparently healthy individuals underscores the importance of functional redundancy in the cytokine network.

## Supporting information

S1 File Danish and English questionnaires for the Danish Blood Donor Study. (PDF)

S2 File Specific c-aAb signals in human IgG pool. An IgG pool derived from 20,000 Danish blood donors was serially diluted and pre-incubated with 100 nM of cytokine prior to incubation with cytokine-conjugated MagPlex beads, as described in the materials and methods section. Data represent average percentage MFI signal displacement with SD, and are representative of experiments on 3 separate IgG pools.
(TIF)

S3 File Univariate analyses of epidemiological variables as predictors of continuous c-aAb MFI signals. Using a series of Wilcoxon rank-sum tests, the association of continuous c-aAb MFI signals to age, sex, obesity and smoking status (all expressed as dichotomous variables, panels A-D) respectively was determined. For the dichotomous variables, being above the mean age of 39.9 years, female sex, active smoking and obesity were defined as “1”. MFI data from all 8,972 participants was included, and results are presented as MFI medians with interquartile range. * denotes a *p* value of < 0.05, **p < 0.01, ***p ≤ 0.001.
(TIF)

S4 File Multivariate analyses of epidemiological variables as predictors of continuous c-aAb MFI signals. Multivariate logistic regression analysis was used to investigate the predictors of detectable levels of individual c-aAb. Age, sex, obesity, smoking and CRP were used as independent variables and high levels of c-aAb as the dependent variable. For dichotomous variables high levels of c-aAb, female sex, active smoking and obesity were defined as “1”. Data are presented as OR with 95% confidence interval for age, sex, obesity, and smoking (panels A-D) as predictors of positive levels of c-aAb. * denotes a *p* value of < 0.05, **p < 0.01, and *** *P* ≤ 0.001.
(TIF)
