# Supplementary figures and images for: Prevalence and correlation of cytokine-specific autoantibodies with epidemiological factors and C-reactive protein in 8,972 healthy individuals: Results from the Danish Blood Donor Study
Source: PLoS One. 2017 Jun 30;12(6):e0179981. doi: 10.1371/journal.pone.0179981 (PMC5493339; doi:10.1371/journal.pone.0179981)

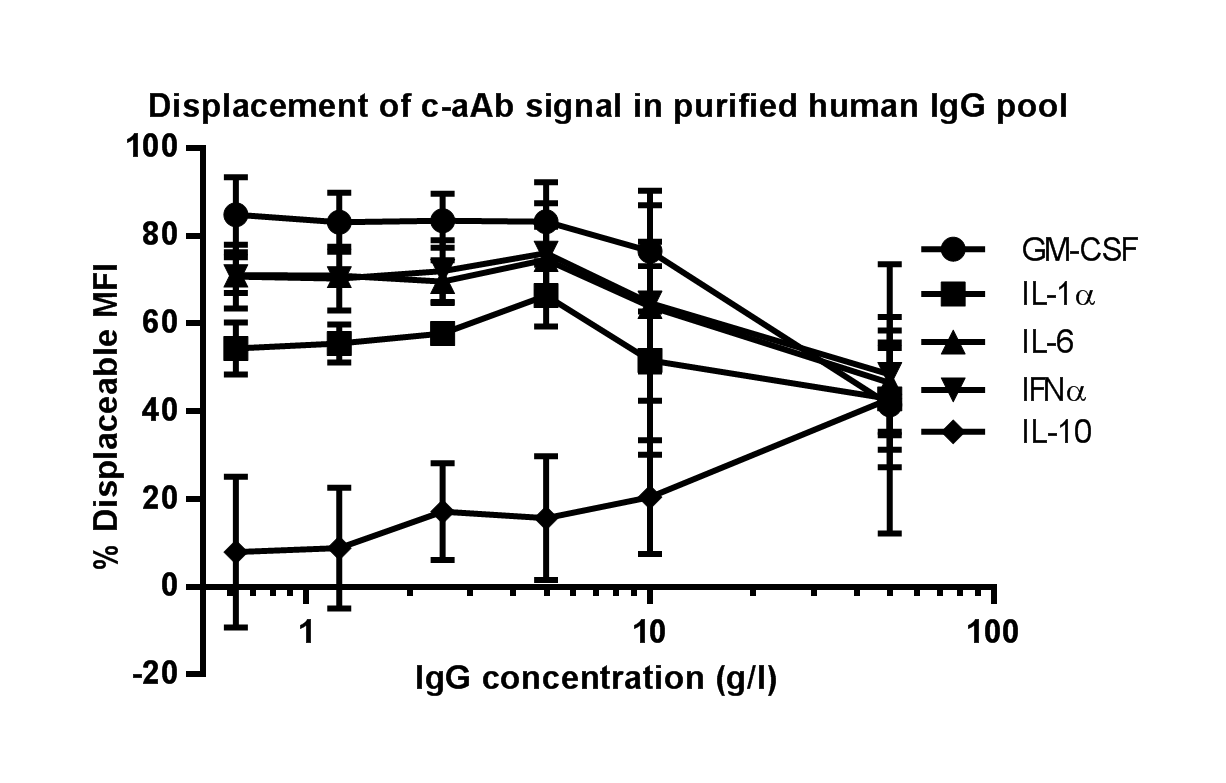

Supplement: S2 File — An IgG pool derived from 20,000 Danish blood donors was serially diluted and pre-incubated with 100 nM of cytokine prior to incubation with cytokine-conjugated MagPlex beads, as described in the materials and methods section. Data represent average percentage MFI signal displacement with SD, and are representative of experiments on 3 separate IgG pools. (TIF) [file pone.0179981.s002.tif]

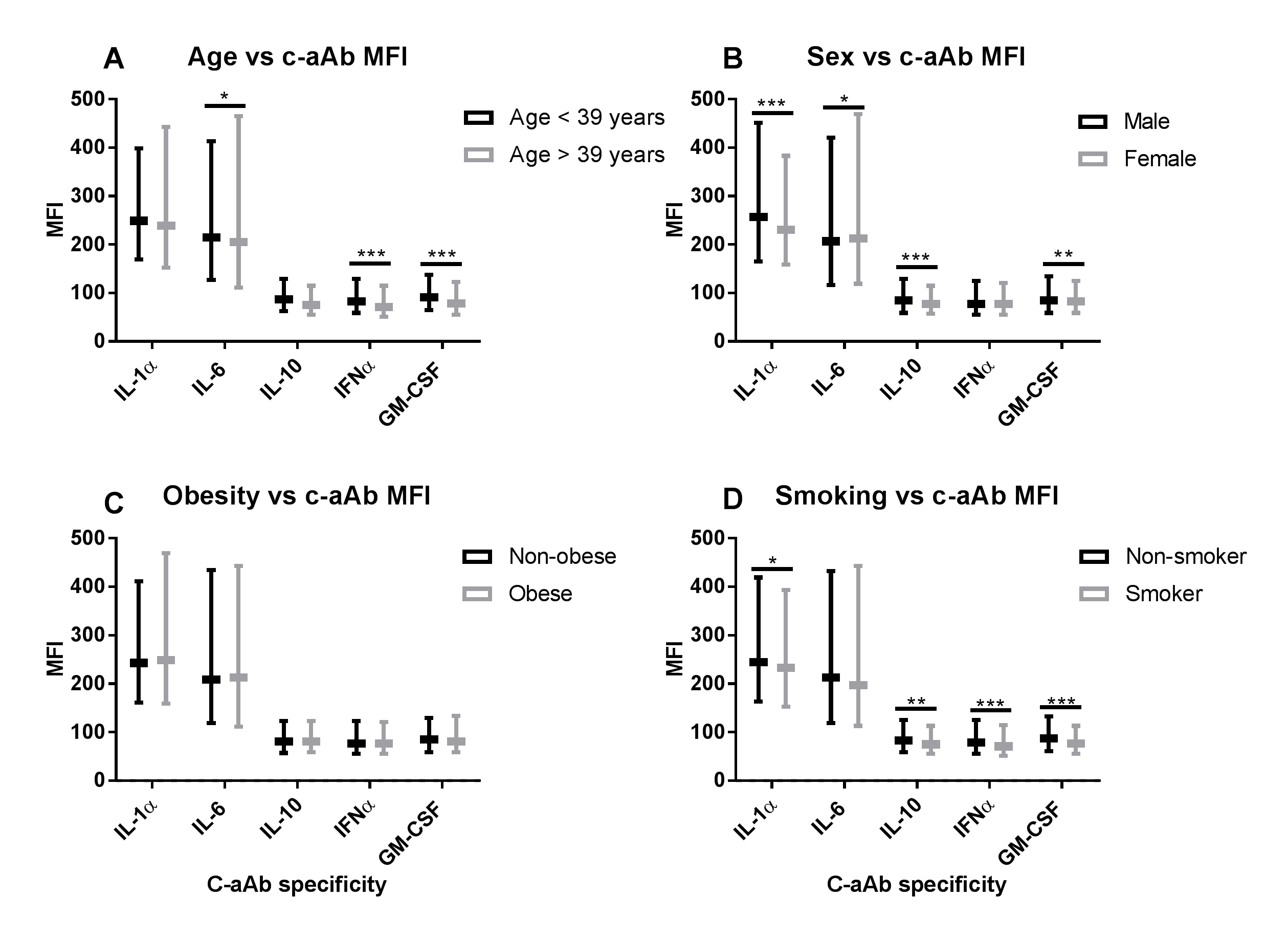

Supplement: S3 File — Using a series of Wilcoxon rank-sum tests, the association of continuous c-aAb MFI signals to age, sex, obesity and smoking status (all expressed as dichotomous variables, panels A-D) respectively was determined. For the dichotomous variables, being above the mean age of 39.9 years, female sex, active smoking and obesity were defined as “1”. MFI data from all 8,972 participants was included, and results are presented as MFI medians with interquartile range. * denotes a p value of < 0.05, **p < 0.01, ***p ≤ 0.001. (TIF) [file pone.0179981.s003.tif]

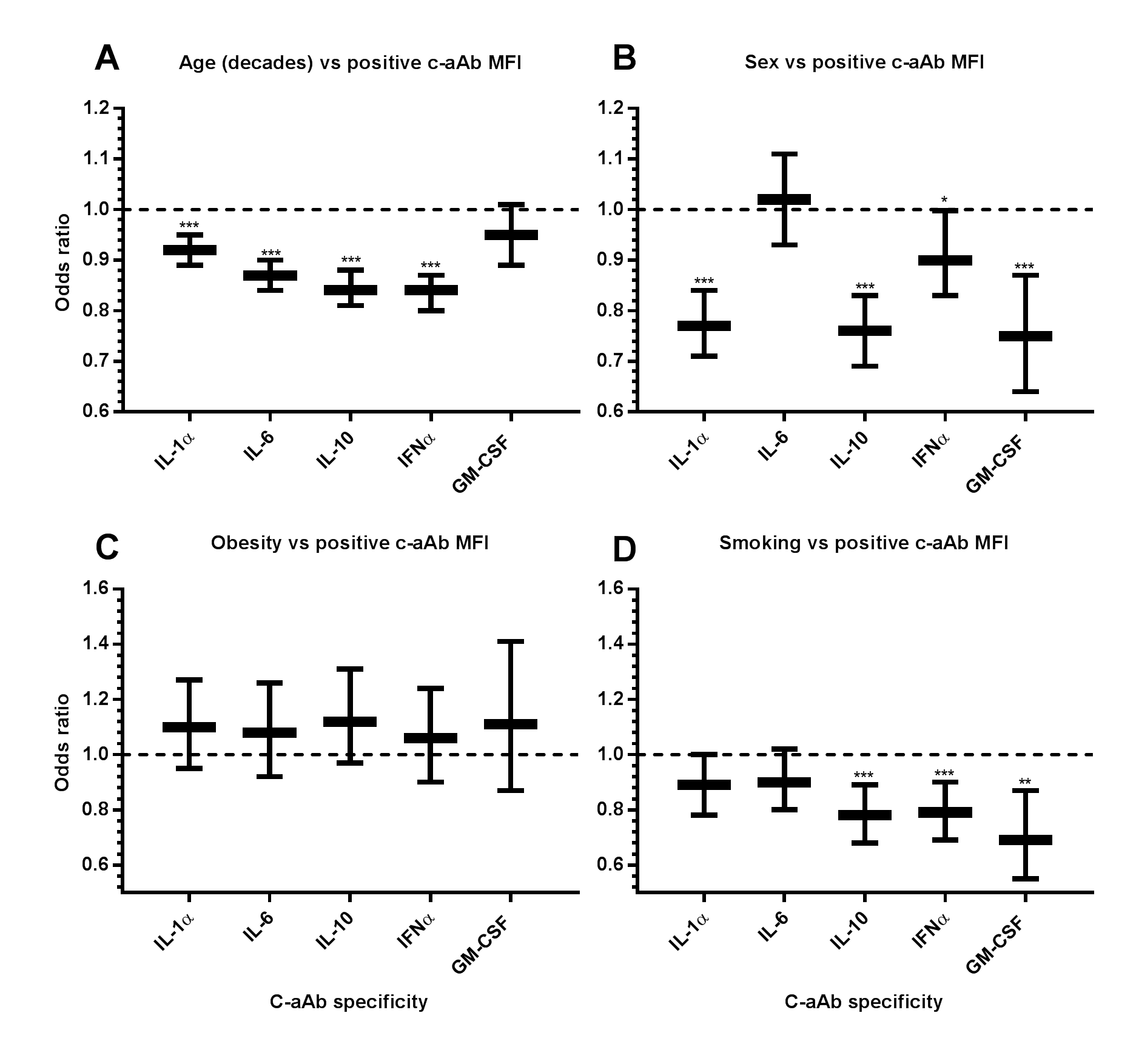

Supplement: S4 File — Multivariate logistic regression analysis was used to investigate the predictors of detectable levels of individual c-aAb. Age, sex, obesity, smoking and CRP were used as independent variables and high levels of c-aAb as the dependent variable. For dichotomous variables high levels of c-aAb, female sex, active smoking and obesity were defined as “1”. Data are presented as OR with 95% confidence interval for age, sex, obesity, and smoking (panels A-D) as predictors of positive levels of c-aAb. * denotes a p value of < 0.05, **p < 0.01, and *** P ≤ 0.001. (TIF) [file pone.0179981.s004.tif]
